# Radiation Oncology Device Approval in the United States and Canada

**DOI:** 10.7759/cureus.4351

**Published:** 2019-04-01

**Authors:** Craig A Beers, Wendy L Smith, Sarah Weppler, Colleen Schinkel, Harvey Quon

**Affiliations:** 1 Radiation Oncology, Cumming School of Medicine, University of Calgary, Calgary, CAN; 2 Medical Physics, University of Calgary, Calgary, CAN; 3 Radiation Oncology, Tom Baker Cancer Centre, Calgary, CAN

**Keywords:** radiation oncology, medical device, health canada, fda

## Abstract

Background

Medical devices are a crucial component in the field of radiation oncology. The review and licensing of radiation oncology devices (RODs) is managed on a national basis in Canada by Health Canada and in the United States by the Food and Drug Administration (FDA). The purpose of this study was to examine differences in ROD licensing timelines between Health Canada and the FDA that may impact the ability of Canadians to access the most up-to-date radiation oncology care.

Methods

A list of ROD was compiled by searching keywords, manufacturers, and proprietary device names in the publicly accessible Canadian Medical Devices Active Licence Listing (MDALL) and the American Establishment Registration & Device Listing and the 510(k) Premarket Notification database. ROD licensing dates were then obtained through both databases. ROD were included if they were licensed in both countries.

Results

A total of 51 RODs were included in this study and it was found that 71% (36/51) were issued licenses for sale in the United States before Canada, at a mean of 506 days sooner (median [IQR] = 282 [326.5]). No trends in licensing dates were found by stratifying devices by type. Analyses were limited to the date of licensing only, as Health Canada provided no publicly-available information regarding submission milestones such as first submission date for the RODs studied.

Conclusions

The majority of radiation oncology devices examined were licensed for sale in the USA before Canada. Due to the absence of publicly available information regarding initial ROD application date, we cannot evaluate the impact of the approval process on the overall difference in licensing date. Importantly, this research highlights a lack of publicly-available information from Health Canada regarding the medical device approval process for the radiation oncology devices studied herein.

## Introduction

Radiation oncology is a field dependent on the availability of the medical devices, including linear accelerators, brachytherapy technologies, patient imaging and monitoring devices, quality assurance (QA) tools, and treatment planning software [1]. With the rapid evolution of radiation oncology devices (RODs) and technology, there is a constant desire by both patients and health care providers to have the most up-to-date technology in use at Canada’s cancer centers [1]. 

The introduction of a medical device to the Canadian market is a complicated process that is regulated by the Medical Devices Regulations of the Food and Drugs Act and overseen by the Therapeutic Products Directorate through the Medical Device Bureau, both contained within Health Canada [2]. Specifically, the Medical Devices Bureau reviews applications for new medical device licenses and contributes to the policy and development of new medical device regulations [3]. Many radiation oncology devices are additionally regulated in accordance with the Class II Nuclear Facilities and Prescribed Equipment Regulations under the Nuclear Safety and Control Act (NSCA) by the Canadian Nuclear Safety Commission (CNSC) [4].

The United States of America has similar provisions; medical device approvals and regulations are mainly covered by the Federal Food, Drug, and Cosmetic Act (FDCA), while the Center for Devices and Radiological Health (CDRH) of the Food and Drug Administration (FDA) is responsible for regulating medical devices in the US market [5]. RODs, in particular, are governed through the Radiation Control provisions of the Radiation Control for Health and Safety Act of 1968 [6] and the United States Food and Drug Administration’s Center for Devices and Radiological Health (CDRH) is responsible for regulating radiation-emitting electronic products [7].

A recent comparison of oncology drug approval time between Health Canada and the FDA found that on average, the time from submission to Health Canada to approval for oncology drugs is three months longer than the same process at the FDA [8]. Similarly, a comparison of all novel therapeutic agents submitted to Health Canada, the FDA, and the European Medicines Agency (EMA) found that on average the FDA reviewed applications for novel therapeutics 44 to 71 days more quickly than Health Canada or the EMA [9]. The authors also found that among drugs approved in the United States and Canada, 86% were first approved in the United States, and the drugs available a median of 355 days earlier in the United States.

A review of current literature identified no published research similarly comparing approval times for RODs in the USA and Canada. The objective of the project was therefore to compare licensing dates for RODs that are approved for sale in both Canada and the USA.

## Materials and methods

We used only publicly accessible information and data to ensure transparency and reproducibility of our findings. To generate a list of RODs that are currently licensed for sale in Canada, a thorough search of Health Canada’s Medical Devices Active Licence Listing, MDALL (https://health-products.canada.ca/mdall-limh/index-eng.jsp), was performed. Search criteria included but were not limited to: keyword searches (Radiation, Linear Accelerator, LINAC, Brachytherapy, Proton, Gamma), company names (Accuray, Brainlab, Elekta Limited, Philips, Theragenics Corporation, Siemens, Varian Medical Systems, Xoft Inc.), and proprietary device names (Novalis, AlignRT, CyberKnife, Aria, MRIDIAN, TrueBeam). Additionally, vendor and exhibitor lists from the American Association of Physicists in Medicine 2017 Annual Scientific Meeting were used to generate additional MDALL search terms including additional ROD manufacturers (Augmentix, Best Medical Canada, C-Rad Positioning, IRT Systems, IsoAid, Mevion Medical Systems, MIM Software Inc., Mobius Medical Systems, Oncology Systems Limited, Raysearch Laboratories, Scandidos, Sensus Healthcare, Sun Nuclear Corporation, Viewray Incorporated, Vision RT Limited). For each entry, the license device first issue date was abstracted for further analyses. Our search was limited to devices issued licenses between January 1, 2000 and July 2, 2018, in order to capture only contemporary devices currently in use.

Once a list of devices licensed for sale in Canada was generated, each device was searched for in the following United States FDA databases: Establishment Registration & Device Listing (https://www.accessdata.fda.gov/scripts/cdrh/cfdocs/cfRL/rl.cfm) and 510(k) Premarket Notification (https://www.accessdata.fda.gov/scripts/cdrh/cfdocs/cfPMN/pmn.cfm). In order to be included in the study dataset, exact matches were required for device name, version, and applicant/company of manufacture. Any device not matching all three criteria were excluded from the dataset. As with the Canadian database, the license device first issue date for each entry was collected.

Once the database was populated with precisely matched devices found in both American and Canadian databases, entries were stratified into the following categories: general accessories (e.g., patient positioning system, shielding device, etc.), brachytherapy technology, imaging and monitoring accessory, linear accelerator, proton accelerator, quality assurance device (QA), superficial radiotherapy device, and treatment planning/record & verify software. Data were then analyzed to determine differences in licensing dates between the United States and Canada. Date difference data were assessed qualitatively for observable trends between the country of licensing and device category.

## Results

A total of 51 devices matched the inclusion criteria for this study (Supplementary Table 1, see Appendix) and were subcategorized as follows: two general accessories, six brachytherapy technology devices, four imaging and monitoring accessories, 13 linear accelerators, one proton accelerator, seven QA devices, one superficial radiotherapy device, and 17 treatment planning/record & verify software packages. Data from the linear accelerator category can be seen in Table 1. Seventy-four (74) devices were excluded due to a lack of exact match of device name, version, company, or due to being licensed for sale in one country or not the other. A listing of excluded devices can be found in Supplementary Table 2 (see Appendix).

**Table 1:** Licensure Information in Canada and the USA for Linear Accelerators from 2000 to Present

Device name	Company	CAN issue date	USA issue date	Difference in days (USA before CAN)	Difference in days (CAN before USA)
Mridian Linac System	Viewray Incorporated	2-Aug-17	24-Feb-17	159	
Oncor Expression Digital Linear Accelerator	Siemens	28-Aug-06	15-Mar-06	166	
Artiste Mv - Linear Accelerator	Siemens	12-Aug-08	27-Dec-07	229	
Halcyon Medical Linear Accelerator	Varian Medical Systems, Inc	6-Jul-18	27-Jun-17	374	
Tomotherapy Treatment System Tomohd	Accuray	27-Nov-13	29-Aug-12	455	
Radixact Treatment Delivery System	Accuray	2-Oct-17	24-Jun-16	465	
Novalis (Shaped Beam Surgery System)	Brainlab Ag	2-Dec-03	3-Nov-00	1124	
Cyberknife Robotic Radiosurgery System - 1000 Mu/Minute Linear Accelerator	Accuray	16-Aug-06	30-Oct-06		75
Brainscan Stereotactic Radiosurgery System	Brainlab Ag	8-Feb-00	13-Jul-00		156
Leksell Gamma Knife Perfexion	Elekta Instrument Ab	30-Aug-06	5-Mar-07		187
Truebeam	Varian Medical Systems, Inc	2-Dec-10	18-Aug-11		259
Cyberknife M6 Series System	Accuray	23-Oct-13	7-Jan-15		441
Cyberknife Vsi Robotic Radiosurgery System	Accuray	18-Mar-11	26-Oct-12		588

**Figure 1:**
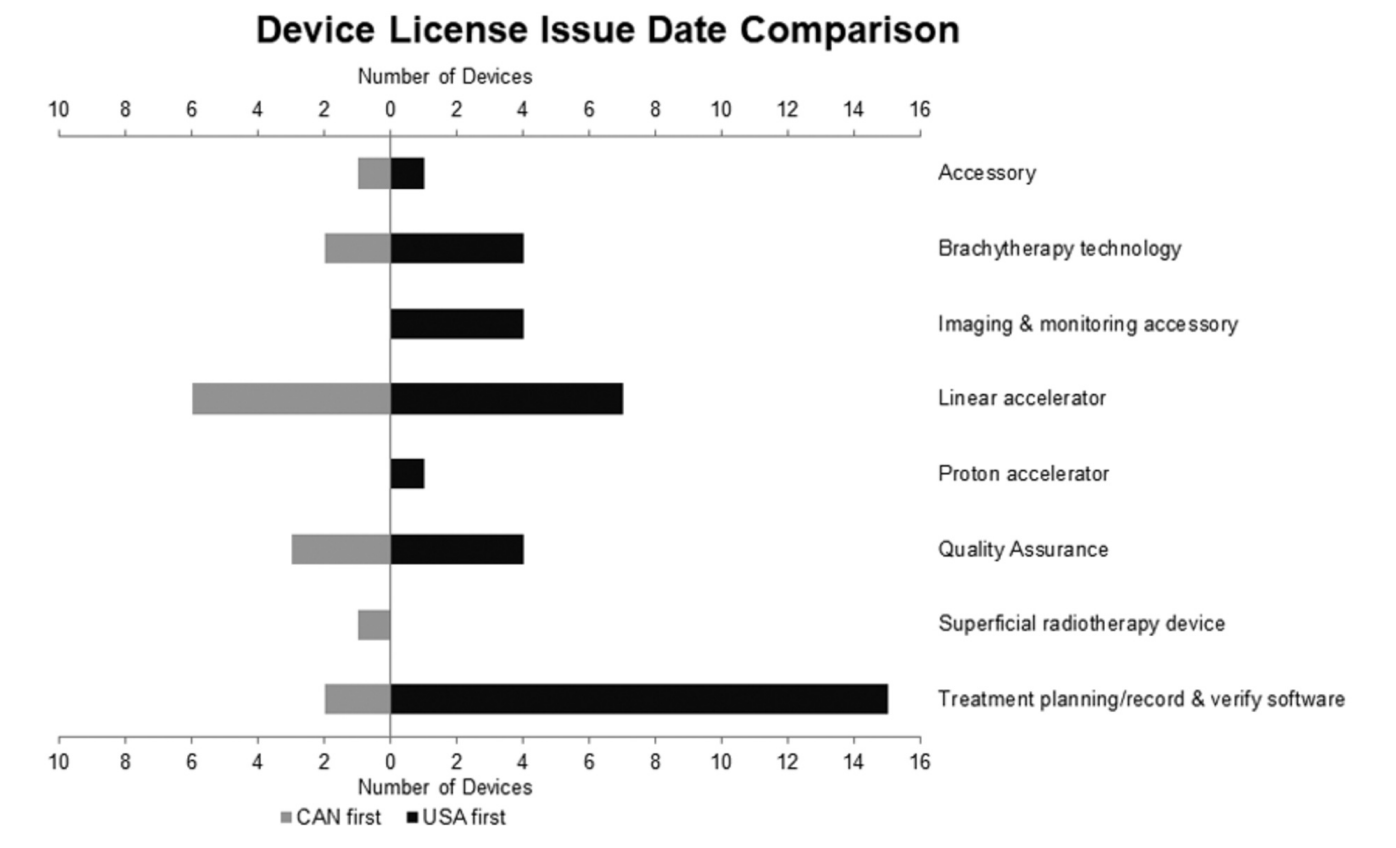
Comparison of Device Licensure Date Between Canada and the USA

In total, 36 (71%) of the 51 devices were licensed for sale in the USA before Canada (Figure 1). Devices were licensed in the USA a mean of 506 days before Canada (median [interquartile range]: 283 [327 days]). Of the 15 devices licensed for sale in Canada before the USA, the mean difference was 571 days (median [interquartile range]: 187 [369 days]). These data can be found in Table 2. 

**Table 2:** Radiation Device Licensure in Canada and the USA (January 2000 – July 2018)

Total number of devices examined	51
Number of devices licensed for sale in the USA before Canada	36
Number of devices licensed for sale in Canada before the USA	15
Devices licensed in the USA before Canada (36/51)	
Mean difference [St.Dev] in licensing date (days)	506 [639.4]
Median [IQR] difference in licensing date (days)	282 [326.5]
Devices licensed in Canada before the USA (15/51)	
Mean difference in [St.Dev] licensing date (days)	572 [1168.1]
Median [IQR] difference in licensing date (days)	187 [369.0]

The results for each category are summarized in Table 3. Of the eight device categories examined, the majority of devices were listed for sale in the USA first in seven categories (88%). The minimum difference in days was found to be 94, while the maximum was 3470 days. The one ROD category where the majority were licensed in Canada first was a superficial radiotherapy device. The single device was licensed in Canada 194 days prior to the USA. Additionally, the devices examined had 24 unique manufacturers. Stratifying these data by the company of manufacture failed to reveal any trend towards favoring licensure in Canada or the United States first; however, it is difficult to draw conclusions from these results as 17/24 (71%) of the examined manufacturers had two devices or fewer included in this dataset. 

**Table 3:** License Date Comparison by Category

Category	Total devices	Number licensed in USA first	Median days difference (IQR)	Number licensed in Canada first	Median days difference (IQR)
Accessory	2	1	244 (n/a)	1	871 (n/a)
Brachytherapy technology	6	4	273 (197)	2	156 (17)
Imaging & Monitoring Accessory	4	4	1124 (477)	0	n/a
Linear Accelerator	13	7	374 (214)	6	187 (209)
Proton Accelerator	1	1	949 (0)	n/a	n/a
Quality Assurance	7	4	229 (198)	3	158 (117)
Superficial RT	1	0	n/a	1	194 (n/a)
Treatment Planning/Record & Verify Software	17	15	283 (290)	2	2477 (380)

## Discussion

Our analysis of licensing date for radiation oncology devices in the United States and Canada from 2000 to 2018 reveals that 71% (36/51) of the devices licensed for sale over that timeframe was licensed in the United States before Canada, a mean of 506 days (1.4 years) earlier. Given that Canada has 43 cancer centers, while the United States has approximately 1500 cancer centers, it is not surprising that companies may choose to go to market in the USA first [10]. From a financial standpoint, the medical device market in the United States is much larger than the Canadian market: SelectUSA, a government program led by the U.S. Department of Commerce, estimates that the United States represents the largest medical device market in the world at US$156 billion (40% of the global medical device market) in 2017 [11], while estimates from the Government of Canada put the Canadian market at US$6.7 billion (6% of the global medical device market) in 2016 [12]. Additionally, we found that 13 out of the 24 companies (54%) included in this study were founded, or had their worldwide headquarters within the USA. This may represent another explanatory variable for our results. No specific trends in licensing date and time were identified by stratifying the devices by category (Table 3).

Licensing fees do not appear to be an explanatory variable for why the majority of RODs are licensed for sale in the United States before Canada. Indeed, medical device costs appear to be much lower for entering the Canadian market: submitting a device for a Medical Device Establishment Licence through Health Canada costs $8,272 CAD [13], applying for the Medical Device Licence Application Review for a Class III device (the majority RODs studied here were Class III) costs $9,881 CAD [14], and the annual Right to Sell Licensed Class II, III, or IV Medical Devices $383 CAD [15]. Fees for the FDA are dependent on whether or not a predicate device has already been approved: If a substantially equivalent predicate legally marketed device exists, a new application through the 510(k) process will cost $10,566 USD ($2,642 USD if the applicant is a Small Business) [16]. If no predicate device is found, a Premarket Approval review will be required at a cost of $310,764 USD (Small business $77,691 USD) [16]. Additionally, there is an Annual Establishment Registration Fee of $4,624 USD [16]. Based on these values, Canadian medical device licensing fees do not appear to be a barrier for bringing RODs to market in Canada.

Unfortunately, without information regarding the initial ROD licensing application date, we are unable to draw any conclusion about whether differences in licensing dates are due to manufacturers applying earlier in one country, or if the delay is in the approvals process itself.

An important issue that we feel should be addressed, is a lack of publicly-available information by Health Canada in the area of medical device licensing. The intended goal of this project was to examine the length of time required for the approval of a medical device in Canada and the United States, from initial submission date to final approval. As previously discussed, this topic has been explored for novel therapeutic agents [9] and specifically for oncology drugs [8]. Unfortunately, the Medical Devices Active Licence Listing (MDALL) database maintained by Health Canada does not publicly provide date of license submission. In comparison, both submission and approval dates are publicly available through the United States FDA databases, the Establishment Registration and Device Listing and 510(k) Premarket Notification database. A comparison of the Canadian and American database output can be seen in Table 4. According to Health Canada’s website, Summary Basis of Decision (SBD) documents are generated for drugs and medical devices for sale in Canada and document regulatory, safety, effectiveness and quality (chemistry and manufacturing) considerations. SBD provide a detailed timeline of submission milestones including submission filing date, completion dates for multiple quality control evaluations, and the Notice of Compliance (NOC) issue date (the NOC is issued following the satisfactory review of a submission for a new drug, and signifies compliance with the Food and Drug Regulations [17-18]). Unfortunately, Health Canada has chosen to only “publish 5-7 SBDs per year for newly licensed Class III and IV devices with novel technology” [19], so information related to dates of submission milestones for the majority of medical devices are not publicly-available. Attempts to acquire additional submission timeline information from the Medical Devices Bureau (MDB) were redirected to request information for each device under the Access to Information Act (https://www.canada.ca/en/health-canada/corporate/contact-us/access-information-privacy-division.html).

**Table 4:** Summary of Equivalent Outputs of the FDA’s 510(k) Premarket Notification Database (left) and Health Canada’s Medical Devices Active Licence Listing (MDALL) FDA, food and drug administration

Category	FDA 510(k)	Health Canada MDALL
Unique Device/Licence identifiers	x	x
Device name	x	x
Device classification	x	x
Manufacturer information	x	x
Licence issue date	x	x
Application received date	x	
Manufacturer contact	x	
Relevant Federal Regulations number	x	
Review panel information	x	
PDF summary of decision	x	
Unique company identifier		x

It is important to discuss the limitations of the research presented here. First, to ensure that comparisons between licensing dates were made as accurately as possible, only RODs with exact matches of the device name, version, and applicant/company of manufacture between Health Canada’s and the FDA’s database were included. However, adherence to this strict inclusion criteria resulted in the exclusion of a number of devices with subtle differences in search criteria. Second, as discussed above, we chose to base the analysis on publicly-available information, and therefore could not perform the intended analysis examining the length of time for the approval process at Health Canada vs. the FDA of the United States. Finally, this research was limited only to North America because of a lack of publicly-available information for medical devices licensed in Europe. The European Database on Medical Devices, Eudamed (https://ec.europa.eu/growth/sectors/medical-devices/market-surveillance_en), is not publicly accessible and therefore data on European radiation oncology devices was not included [20]. 

## Conclusions

In summary, we observed that 71% of radiation oncology devices licensed for sale in both Canada and the United States between 2000 and 2018 were licensed in the United States before Canada, a mean of 506 days sooner. Due to a lack of publicly-available information from Health Canada we were unable to rule out the earlier application as a cause for this discrepancy as opposed to differences in duration of approval processes. This highlights the paucity of publicly available data regarding medical device approvals in Canada which is available in the United States.

## Appendices

Supplementary Table 1

**Table 5:** Radiation Oncology Device Listing and Licensure Date for All Categories

Device name	Classification	Company	CAN issue date	USA issue date	Date difference: USA before CAN	Date difference: CAN before USA
Axxent Radiation Shield Rigid	Accessory	XOFT, INC., A SUBSIDIARY OF ICAD	20-Mar-12	20-Jul-11	244	n/a
Spaceoar System	Accessory	AUGMENIX	5-Feb-16	25-Jun-18	n/a	871
I-seed Model AgX100	Brachytherapy	THERAGENICS CORPORATION	7-Apr-11	3-Jan-11	94	n/a
Axxent Vaginal Applicator Set	Brachytherapy	XOFT, INC., A SUBSIDIARY OF ICAD	6-Feb-09	9-May-08	273	n/a
Radioactive Seed Localization Needle	Brachytherapy	ISOAID L.L.C.	19-Jan-16	5-Dec-14	410	n/a
Advantage I-125 Brachytherapy Seeds	Brachytherapy	ISOAID L.L.C.	4-Dec-03	7-Oct-01	788	n/a
Advantage Pd-103 Brachytherapy Seeds	Brachytherapy	ISOAID L.L.C.	4-Dec-03	8-Apr-04	n/a	126
Axxent Balloon Applicator Kit AB2034	Brachytherapy	XOFT, INC., A SUBSIDIARY OF ICAD	6-Feb-09	16-Jul-09	n/a	160
Exactrac Patient Positioning System	Imaging & Monitoring Accessory	BRAINLAB AG	25-Jan-02	14-Jul-99	926	n/a
Catalyst	Imaging & Monitoring Accessory	C-RAD POSITIONING AB	17-Feb-15	27-Feb-12	1086	n/a
Sentinel, Sp-001	Imaging & Monitoring Accessory	C-RAD POSITIONING AB	30-Apr-14	30-Mar-09	1857	n/a
AlignRT	Imaging & Monitoring Accessory	VISION RT LIMITED	8-Jul-15	6-Jan-06	3470	n/a
Mridian Linac System	Linear Accelerator	VIEWRAY INCORPORATED	2-Aug-17	24-Feb-17	159	n/a
Oncor Expression Digital Linear Accelerator	Linear Accelerator	SIEMENS	28-Aug-06	15-Mar-06	166	n/a
Artiste Mv - Linear Accelerator	Linear Accelerator	SIEMENS	12-Aug-08	27-Dec-07	229	n/a
Halcyon Medical Linear Accelerator	Linear Accelerator	VARIAN MEDICAL SYSTEMS, INC	6-Jul-18	27-Jun-17	374	n/a
Tomotherapy Treatment System Tomohd	Linear Accelerator	ACCURAY	27-Nov-13	29-Aug-12	455	n/a
Radixact Treatment Delivery System	Linear Accelerator	ACCURAY	2-Oct-17	24-Jun-16	465	n/a
Novalis (Shaped Beam Surgery System)	Linear Accelerator	BRAINLAB AG	2-Dec-03	3-Nov-00	1124	n/a
Cyberknife Robotic Radiosurgery System - 1000 Mu/Minute Linear Accelerator	Linear Accelerator	ACCURAY	16-Aug-06	30-Oct-06	n/a	75
Brainscan Stereotactic Radiosurgery System	Linear Accelerator	BRAINLAB AG	8-Feb-00	13-Jul-00	n/a	156
Leksell Gamma Knife Perfexion	Linear Accelerator	ELEKTA INSTRUMENT AB	30-Aug-06	5-Mar-07	n/a	187
Truebeam	Linear Accelerator	VARIAN MEDICAL SYSTEMS, INC	2-Dec-10	18-Aug-11	n/a	259
Cyberknife M6 Series System	Linear Accelerator	ACCURAY	23-Oct-13	7-Jan-15	n/a	441
Cyberknife Vsi Robotic Radiosurgery System	Linear Accelerator	ACCURAY	18-Mar-11	26-Oct-12	n/a	588
Mevion S250 Proton Beam Radiation Therapy System	Proton Accelerator	MEVION MEDICAL SYSTEMS	9-Jan-15	4-Jun-12	949	n/a

Device name	Classification	Company	CAN issue date	USA issue date	Date difference: USA before CAN	Date difference: CAN before USA
Delta4 Phantom+	Quality Assurance	SCANDIDOS AB	18-Sep-15	19-Aug-15	30	n/a
Perfraction	Quality Assurance	SUN NUCLEAR CORPORATION	3-Nov-14	26-Sep-14	38	n/a
Portable Dosimeter TN-RD-90	Quality Assurance	BEST MEDICAL CANADA	25-Oct-10	28-Aug-09	423	n/a
Profiler 2	Quality Assurance	SUN NUCLEAR CORPORATION	7-Feb-08	22-Nov-06	442	n/a
3D Scanner	Quality Assurance	SUN NUCLEAR CORPORATION	29-Sep-10	1-Oct-10	n/a	2
IGM Integral Quality Monitor	Quality Assurance	IRT SYSTEMS GMBH	14-Sep-16	20-Oct-16	n/a	36
Mobius3d	Quality Assurance	MOBIUS MEDICAL SYSTEMS, LP	17-Jan-13	24-Jun-14	n/a	523
Srt-100 Superficial Radiation Therapy System	Superficial Radiotherapy	SENSUS HEALTHCARE	1-Nov-12	14-May-13	n/a	194
Integrity	Treatment Planning/Record & Verify Software	ELEKTA LIMITED	27-Jan-12	16-Dec-11	42	n/a
Aria Radiation Therapy Management	Treatment Planning/Record & Verify Software	VARIAN MEDICAL SYSTEMS, INC	21-May-14	4-Apr-14	47	n/a
Iplan! (Stereotactic Planning Software)	Treatment Planning/Record & Verify Software	BRAINLAB AG	8-Dec-04	22-Oct-04	47	n/a
Mobile Mim	Treatment Planning/Record & Verify Software	MIM SOFTWARE INC	24-Mar-11	4-Feb-11	48	n/a
Accuray Precision Treatment Planning System	Treatment Planning/Record & Verify Software	ACCURAY	30-Oct-17	8-Jun-17	144	n/a
iViewDose	Treatment Planning/Record & Verify Software	ELEKTA LIMITED	27-May-16	11-Dec-15	168	n/a
Eclipse Treatment Planning System V13.0	Treatment Planning/Record & Verify Software	VARIAN MEDICAL SYSTEMS, INC	4-Sep-14	7-Feb-14	209	n/a
Raystation 5	Treatment Planning/Record & Verify Software	RAYSEARCH LABORATORIES AB (PUBL)	14-Nov-16	8-Mar-16	251	n/a
Variseed 9.0	Treatment Planning/Record & Verify Software	VARIAN MEDICAL SYSTEMS, INC	1-Feb-16	8-May-15	269	n/a
OnQ rts Software	Treatment Planning/Record & Verify Software	ONCOLOGY SYSTEMS LIMITED	27-Sep-13	28-Dec-12	273	n/a
mobileMOSFET Wireless Dosimetry System	Treatment Planning/Record & Verify Software	BEST MEDICAL CANADA	13-Apr-05	25-Jun-04	292	n/a
Eclipse Treatment Planning System V15.5	Treatment Planning/Record & Verify Software	VARIAN MEDICAL SYSTEMS, INC	6-Jul-18	15-Aug-17	325	n/a
Eclipse Treatment Planning System V13.5	Treatment Planning/Record & Verify Software	VARIAN MEDICAL SYSTEMS, INC	28-Aug-15	7-Aug-14	386	n/a
Variseed 7.0	Treatment Planning/Record & Verify Software	VARIAN MEDICAL SYSTEMS, INC	31-Jan-03	12-Jul-01	568	n/a
Eclipse Treatment Planning System V6.5	Treatment Planning/Record & Verify Software	VARIAN MEDICAL SYSTEMS, INC	7-Jun-04	2-May-01	1132	n/a
Variseed 7.1	Treatment Planning/Record & Verify Software	VARIAN MEDICAL SYSTEMS, INC	11-Feb-04	21-May-04	n/a	100
Pinnacle 3 Radiation Therapy Planning System	Treatment Planning/Record & Verify Software	PHILIPS MEDICAL SYSTEMS (CLEVELAND), INC	28-Feb-00	14-Jun-13	n/a	4855

Supplementary Table 2

**Table 6:** Excluded Devices

Number	Device name	Classification	Company	Canada issue date	USA received date
1	ABC MOUTHPIECE AND FILTER KIT	ACCESSORY	AKTINA MEDICAL	01-Jun-18	n/a
2	BARD BRACHYSTAR SEED IMPLANT NEEDLE	ACCESSORY	CR BARD	30-May-12	n/a
3	LOGICIEL EPIGRAY	ACCESSORY	DOSISOFT	02-Feb-16	n/a
4	ACTIVE BREATHING CO-ORDINATOR SYSTEM	ACCESSORY	ELEKTA LIMITED	22-Jul-05	n/a
5	STEREOTACTIC COLLIMATOR	ACCESSORY	ELEKTA LIMITED	21-Jul-09	n/a
6	AGILITY	ACCESSORY	ELEKTA LIMITED	15-Nov-12	n/a
7	MOBIUS VERIFICATION PHANTOM	ACCESSORY	MOBIUS MEDICAL SYSTEMS, LP	29-Apr-15	n/a
8	DELTA4 DISCOVER	ACCESSORY	SCANDIDOS AB	07-Feb-18	n/a
9	QED	ACCESSORY	SUN NUCLEAR CORPORATION	10-Jan-08	n/a
10	ISORAD	ACCESSORY	SUN NUCLEAR CORPORATION	10-Jan-08	n/a
11	RF IVD2	ACCESSORY	SUN NUCLEAR CORPORATION	16-Jan-08	n/a
12	EDGE DETECTOR	ACCESSORY	SUN NUCLEAR CORPORATION	07-Feb-08	n/a
13	SNC125C	ACCESSORY	SUN NUCLEAR CORPORATION	09-Nov-11	n/a
14	OPTICAL SURFACE MONITORING SYSTEM (OSMS)	ACCESSORY	VISION RT LIMITED	29-Jul-16	n/a
15	AXXENT SURFACE APPLICATOR SET	ACCESSORY	XOFT, INC., A SUBSIDIARY OF ICAD	07-Oct-10	n/a
16	IODINE 125 SEEDS	BRACHYTHERAPY	BEST MEDICAL INTERNATIONAL	26-Jun-01	n/a
17	IRIDIUM 192 IN NYLON RIBBONS	BRACHYTHERAPY	BEST MEDICAL INTERNATIONAL	26-Jun-01	n/a
18	PALLADIUM 103	BRACHYTHERAPY	BEST MEDICAL INTERNATIONAL	26-Jun-01	n/a
19	GOLD 198	BRACHYTHERAPY	BEST MEDICAL INTERNATIONAL	26-Jun-01	n/a
20	BRACHYTHERAPY NEEDLES - NON STAINLESS STEEL	BRACHYTHERAPY	BEST MEDICAL INTERNATIONAL	10-Sep-01	n/a
21	BRACHYTHERAPY NEEDLES - STAINLESS STEEL	BRACHYTHERAPY	BEST MEDICAL INTERNATIONAL	10-Sep-01	n/a
22	BRACHYTHERAPY IMPLANT/APPLICATOR KITS	BRACHYTHERAPY	BEST MEDICAL INTERNATIONAL	10-Sep-01	n/a
23	BRACHYTHERAPY MARKER KITS	BRACHYTHERAPY	BEST MEDICAL INTERNATIONAL	26-Sep-01	n/a
24	STYLETS FOR BRACHYTHERAPY NEEDLES	BRACHYTHERAPY	BEST MEDICAL INTERNATIONAL	31-May-06	n/a
25	PD-103 SOURCES PRELOADED	BRACHYTHERAPY	BEST MEDICAL INTERNATIONAL	26-Jul-06	n/a
26	I-125 SOURCES PRELOADED	BRACHYTHERAPY	BEST MEDICAL INTERNATIONAL	26-Jul-06	n/a
27	POLYACETAL BRACHYTHERAPY KITS	BRACHYTHERAPY	BEST MEDICAL INTERNATIONAL	08-Oct-09	n/a
28	BRACHYTHERAPY STANDARD TEMPLATES	BRACHYTHERAPY	BEST MEDICAL INTERNATIONAL	21-Feb-12	n/a
29	BRACHYTHERAPY DISPOSABLE TEMPLATE KITS	BRACHYTHERAPY	BEST MEDICAL INTERNATIONAL	21-Feb-12	n/a
30	BEST MULTI-LUMEN BALLOON APPLICATOR FOR BRACHYTHERAPY	BRACHYTHERAPY	BEST MEDICAL INTERNATIONAL	09-Dec-14	n/a
31	ANORECTAL (AR) APPLICATOR	BRACHYTHERAPY	BIONIX RADIATION THERAPY	04-Aug-17	n/a
32	ESOPHAGEAL APPLICATOR (E-APP)	BRACHYTHERAPY	BIONIX RADIATION THERAPY	04-Aug-17	n/a
33	BRACHYTHERAPY KIT	BRACHYTHERAPY	ISOAID L.L.C.	03-Jun-11	n/a
34	GUIDE FOR BRACHYTHERAPY NEEDLE	BRACHYTHERAPY	ISOAID L.L.C.	18-Jun-13	n/a
35	ISOLOADER SYSTEM	BRACHYTHERAPY	THERAGENICS CORPORATION	27-Nov-01	n/a
36	ISOCARTRIDGE	BRACHYTHERAPY	THERAGENICS CORPORATION	22-Apr-02	n/a
37	BRACHYTHERAPY NEEDLE	BRACHYTHERAPY	THERAGENICS CORPORATION	19-Sep-02	n/a
38	BRACHYTHERAPY SYNTHETIC ABSORBABLE, BRAIDED AND NON BRAIDED PLACEMENT SLEEVES	BRACHYTHERAPY	THERAGENICS CORPORATION	06-Feb-03	n/a
39	BRACHYTHERAPY NEEDLES	BRACHYTHERAPY	THERAGENICS CORPORATION	18-Jun-03	n/a
40	BRACHYTHERAPY NEEDLE SETS	BRACHYTHERAPY	THERAGENICS CORPORATION	18-Dec-03	n/a
41	GOLD FIDUCIARY MARKERS	BRACHYTHERAPY	THERAGENICS CORPORATION	18-Feb-08	n/a
42	BRACHYTHERAPY APPLICATOR KITS	BRACHYTHERAPY	THERAGENICS CORPORATION	30-Jan-09	n/a
43	PROSTATE SEEDING KITS (THERALOAD, THERASEED, THERASTRAND, THERASLEEVE)	BRACHYTHERAPY	THERAGENICS CORPORATION	09-Jul-10	n/a
44	THERASEED PD-103 DEVICE	BRACHYTHERAPY	THERAGENICS CORPORATION	25-Jan-11	n/a
45	AXXENT ELECTRONIC BRACHYTHERAPY SYSTEM	BRACHYTHERAPY	XOFT, INC., A SUBSIDIARY OF ICAD	06-Feb-09	n/a
46	MLCI2	LINAC	ELEKTA LIMITED	03-Apr-09	n/a
47	ELEKTA MEDICAL LINEAR ACCELERATOR	LINAC	ELEKTA LIMITED	26-Aug-10	n/a
48	CHECKMATE 2	QA	SUN NUCLEAR CORPORATION	16-Jan-08	n/a
49	DAILY QA 3	QA	SUN NUCLEAR CORPORATION	16-Jan-08	n/a
50	DAILY QA 3 RF	QA	SUN NUCLEAR CORPORATION	16-Jan-08	n/a
51	TOMODOSE	QA	SUN NUCLEAR CORPORATION	07-Feb-08	n/a
52	SRS PROFILER	QA	SUN NUCLEAR CORPORATION	31-Dec-08	n/a
53	MAPCHECK 2	QA	SUN NUCLEAR CORPORATION	31-Dec-08	n/a
54	IC PROFILER	QA	SUN NUCLEAR CORPORATION	24-Feb-09	n/a
55	PC ELECTROMETER	QA	SUN NUCLEAR CORPORATION	08-Jun-09	n/a
56	ARCCHECK	QA	SUN NUCLEAR CORPORATION	19-Aug-09	n/a
57	3DVH ANALYSIS SOFTWARE	QA	SUN NUCLEAR CORPORATION	16-Jun-10	n/a
58	1D SCANNER	QA	SUN NUCLEAR CORPORATION	11-Apr-11	n/a
59	STEREOPHAN	QA	SUN NUCLEAR CORPORATION	16-Jul-13	n/a
60	SNC MACHINE	QA	SUN NUCLEAR CORPORATION	15-May-15	n/a
61	DOSECHECK	QA	SUN NUCLEAR CORPORATION	19-May-16	n/a
62	MAPCHECK 3	QA	SUN NUCLEAR CORPORATION	19-Sep-17	n/a
63	SUNCHECK	QA	SUN NUCLEAR CORPORATION	18-Dec-17	n/a
64	SRS MAPCHECK	QA	SUN NUCLEAR CORPORATION	31-May-18	n/a
65	RADIATION QUALITY ASSURANCE SOFTWARE (DOSELAB)	SOFTWARE	MOBIUS MEDICAL SYSTEMS, LP	26-Mar-11	n/a
66	SRT-100 VISION SUPERFICIAL RADIATION THERAPY SYSTEM	SUPERFICIAL RT	SENSUS HEALTHCARE	30-Mar-16	n/a
67	RADPOS 4-D IN-VIVO DOSIMETRY SYSTEM TN-RD-140	TREATMENT PLANNING	BEST MEDICAL CANADA	30-Dec-10	n/a
68	MIM (SOFTWARE PACKAGE)	TREATMENT PLANNING	MIM SOFTWARE INC	03-Oct-05	n/a
69	MIMVIEWER	TREATMENT PLANNING	MIM SOFTWARE INC	05-Jan-09	n/a
70	RIT 113 RADIATION THERAPY DOSIMETRY SOFTWARE	TREATMENT PLANNING	RADIOLOGICAL IMAGING TECHNOLOGY	26-Apr-06	n/a
71	INVERSEARC 1.0	TREATMENT PLANNING	RAYSEARCH LABORATORIES AB (PUBL)	01-Jun-15	n/a
72	DELTA 4PT	TREATMENT PLANNING	SCANDIDOS AB	26-Mar-13	n/a
73	HEXAMOTION	TREATMENT PLANNING	SCANDIDOS AB	21-Jan-14	n/a
74	PLANIQ	TREATMENT PLANNING	SUN NUCLEAR CORPORATION	23-Mar-15	n/a
